# Upper and lower musculoskeletal back pain, stress, physical activity, and organisational work support: An exploratory study of police investigative interviewers

**DOI:** 10.1177/20551029221146396

**Published:** 2022-12-16

**Authors:** Lillis Rabbing, Brita Bjørkelo, Eva Langvik

**Affiliations:** 1 6274 Norwegian Police University College, Oslo, Norway; 2 Department of Psychology, 8018 Norwegian University of Science and Technology, NTNU, Trondheim, Norway

**Keywords:** pain, social support, physical activity, psychological distress, quantitative methods, stress

## Abstract

Police investigative interviewers in special victims’ units have particularly stressing work conditions. Being few in numbers, with highly specialised competence, the health and well-being of this workgroup are key. This study explores the prevalence of muscular lower and upper back pain and stress and associations with physical activity and organisational work support among 77 police investigators. The police investigative interviewers reported high levels of physical activity. Compared to other police employees, they reported similar levels of musculoskeletal back pain, higher levels of upper back pain, and higher levels of stress. Physical activity was not related to musculoskeletal back pain. In the regression analysis, musculoskeletal back pain was negatively associated with organisational work support. Limitations due to low statistical power and a cross-sectional design apply. However, the study provides interesting insight into the prevalence of musculoskeletal back pain and its association with organisational work support and stress among police employees.

## Introduction

Acute and enduring stress in the police is an inevitable part of the police profession (Violanti et al., 2017; Webster, 2014). Despite the systematic finding that organisational factors contribute to ill health to a larger extent than work tasks (Purba and Demou, 2019; Shane, 2020; Violanti et al., 2017), police stress research has mainly emphasised so-called traditional types of police work and acute types of stress in operational settings (Kroes, 1976; Larsen et al., 2019; Rabbing et al., 2022). A growing literature, however, investigates the impact of the work organisation and strain on police investigators (Baeriswyl et al., 2016; Fyhn et al., 2016).

Chronic and untreated stress in combination with insufficient resources among police investigative interviewers in special victim units may pose a threat towards the legal protection of both victims and those accused of a crime. Being relatively few in numbers compared to other groups within the police (e.g., operative or other criminal investigation units), with highly specialised competence (Powell et al., 2010), high staff turnover (Aarons et al., 2004), the health and well-being of this working group are key. Association between basic working condition factors (e.g., psychological, social, organisational, and mechanical) and employees’ health in the form of musculoskeletal complaints and pain is well-documented (Armon et al., 2010; Nielsen et al., 2021). However, the mechanisms underpinning physical activity on musculoskeletal back pain are unclear (Smith et al., 2019) and have rarely been studied among police employees (Larsen et al., 2018; Nabeel et al., 2007). This study explores the prevalence of muscular lower and upper back pain, stress, and associations with physical activity and organisational work support among police investigator interviewers.

### Factors influencing the health of police investigative interviewers

According to the Job Demands-Resources model (JD-R model) (Bakker and Demerouti, 2007), job demands and job resources interplay in the development of health impairment and well-being. The theoretical framework has been widely applied in the police context (Hu et al., 2016; Lambert et al., 2018; Raper et al., 2019) and is used as a backdrop for this study.

Job demands are defined as “physical, psychological, social, or organisational aspects of the job that require sustained physical and/or psychological (cognitive and emotional) effort or skills and are therefore associated with certain physiological and/or psychological costs” (Bakker and Demerouti, 2007, p. 312). Musculoskeletal complaints and pain are consistently related to job demands (Armstrong et al., 1993; Huang et al., 2002). In “traditional” police stress research, low back pain is usually understood as an outcome of demanding work tasks such as car driving, wearing body armour and personal protective equipment, lifting, carrying (Douma et al., 2017; Larsen et al., 2018; Nahit et al., 2003; von dem Knesebeck et al., 2005), and the use of force in challenging cases of arrest (Henze et al., 2022). A growing part of police work, however, is dedicated to working tasks with impaired work condition such as organisational and interagency tensions (Fortune et al., 2018; Powell et al., 2013; Wilson-Kovacs, 2021). A study among newly employed workers from 12 diverse occupational groups found psychosocial stress to be associated with a doubled risk for musculoskeletal pain (Nahit et al., 2003).

Musculoskeletal back pain is relatively frequent among police officers (Colgan et al., 2019; Larsen et al., 2018), especially lower back pain, reported by about 30% (Larsen et al., 2018). However, pain in the upper back regions is also common (Cho et al., 2014; Rhee et al., 2015). Larsen et al. (2019) found that police workplaces characterised by low control and high social support in addition to active and high strained jobs, as well as high demands, were related to increased impaired health in the form of musculoskeletal pain. Known demanding job characteristics for police investigative interviewers at special units are inadequate recognition of specialised skills from supervisors, high emotional demands, workload, and interagency tensions (Jakobsen et al., 2017; Perez et al., 2010; Powell et al., 2013; Risan, 2017; Risan et al., 2016a, 2016b).

In the JD-R model, job resources describe the motivational part of the process, such as organising tasks in a way contributing to goal achievement and alleviating job demands in addition to stimulating personal growth, learning, and development (Bakker and Demorouti, 2007). The causal assumptions where job characteristics lead to employee well-being have been supported by a meta-analysis by Lesener and colleagues (2019). An affective commitment, i.e., pride in the organization, internalisation of goals, and acceptance of core values, often lead to psychological bonds with the organisation (Hu et al., 2016). Employees’ alignment with their organisation’s strategic objectives is important in the development of adverse health outcomes and may reduce psychological strain (Biggs et al., 2014; Raper et al., 2019). Long-term positive leadership climate (Engel et al., 2018) and organisational structure (Lambert et al., 2018) often result in higher affective commitment, and fair and supportive leadership is identified as a protective factor against burnout among police employees (Sørengaard and Langvik, 2022). The way to improve the work demands for investigative interviewers in the police is thus not only a question of decreasing turnover and increasing job rotation, but also modifying factors linked to the organisational stressors (Raper et al., 2019) and that the demands are offset by the resources (Powell et al., 2013).

Physical activity and organisational work support from leaders and colleagues are job resources with motivational qualities which, according to the JD-R model, may buffer harmful effects on health (Geneen et al., 2017; Strauss et al., 2021; Wolter et al., 2019), the effect of job demands on emotional exhaustion (Santa Maria et al., 2018, 2019), and has an inverse relation with stress (Kula, 2017; Smoktunowicz et al., 2015; Tyagi and Dhar, 2014). Physical fitness is a protective factor concerning workplace ill-health (Kelly, 2020; Rayson, 2000), while poor physical health is associated with low well-being and musculoskeletal pain (Baker et al., 2020; Nielsen et al., 2021; Wolter et al., 2019). People working under stress are likely to smoke more, exercise less, and have an unhealthy diet (Galanis et al., 2019). Exercise and physical activity prevent the degeneration of joints and muscles (Karamanidis and Arampatzis, 2005; Larsen et al., 2019; Van Hecke et al., 2013), improve mental health (Carek et al., 2011; Chan et al., 2019), and negative emotions (Ligeza et al., 2019; Long et al., 2021). The strong emphasis on the importance of physical health in the police is evident in recruitment procedures across national contexts regardless of envisioned future police work tasks (see e.g., Bjørgo and Damen, 2020; Henze et al., 2022).

Gerber et al. (2016, 2013) and Chase et al. (2021) found that police employees reporting high leisure-time exercise levels exhibited fewer health problems. They were also less likely to report high levels of perceived stress (Jonsdottir et al., 2010; Mucke et al., 2018), and reported better sleep (Beak et al., 2021; Fekedulegn et al., 2018). The results from the study by Schilling et al. (2020) showed lowered physiological stress reactivity to acute work stress and physiological recovery for police officers with higher levels of cardiorespiratory fitness. Further, Lentz et al. (2019) identified associations between aerobic fitness and decreased risk of injury among emergency responders. Still, the direct link between physical activity and reduced musculoskeletal back pain remains partly unexplored (Dzakpasu et al., 2021; Schilling et al., 2020; Sitthipornvorakul et al., 2011), among police employees in general, and police investigative interviewers especially.

Hence, there is a need to expand studies in the police. Musculoskeletal complaints and pain are not solely outcomes of physically demanding “traditional” police work tasks but also related to work environment factors (Larsen et al., 2019). We know less about job demands and resources in police work characterised as physically sedentary (e.g., investigative) and psychologically demanding (e.g., interviewing). In the present study we explore associations between stress, physical activity, organisational work support, and musculoskeletal back pain among investigative interviewers in the police.

### Aim of study

Police investigative interviewers are numerically few and a highly skilled workgroup (Powell et al., 2010) known to have high work demands (Burns et al., 2008; Fortune et al., 2018) and high staff turnover (Aarons et al., 2004). The most stressful demands are working factors such as caseload, lack of leader recognition and support, and emotional commitment (Powell et al., 2013). Musculoskeletal back pain is a frequent form of workplace ill-health associated with psychosocial working environment factors, while physical activity and organisational work support are known job resources that may buffer the impact of the health impairing factors. This study explores the health and well-being of police investigative interviewers by investigating the prevalence of work-related ill health outcomes of particular relevance, namely musculoskeletal upper and lower back pain and stress, and how they are associated with physical activity, and organisational work support. Based on the reviewed literature, we outlined the following hypothesis:

H1 *Musculoskeletal pain is negatively associated with physical activity*

H2 *Musculoskeletal pain is negatively associated with organisational work support*

H3 *Musculoskeletal pain is positively associated with stress*

## Method

### Participants and procedures

Inclusion criteria were police officers in the Norwegian Police Service working with investigative interviews as a part of the prosecution process. This sampling procedure is best described as purposive criterion sampling that is “reviewing and studying ‘all cases that meet some predetermined criterion of importance’” (Patton, 2002, p. 238, cited in Suri, 2011). This procedure is applied both in qualitative and quantitative studies (Cronje and Vilakazi, 2020)**.** For reasons of confidentiality, an anonymous paper-and-pen survey, with stamped and addressed envelopes for return use, was distributed by postal mail primo 2020. The recipients were 100 police investigators working in the largest six of the total 12 police districts in Norway. One more police district was invited but did not respond to our request. 77 of the questionnaires were returned, with 49% of the participants being female (20, 8% did not report their gender). Age was operationalised as a categorical variable (*n*, %); 18–29 (27, 35%), 30–39 (21, 27%), 40–49 (21, 27%), 50–59 (8, 10%) with 30–39 as median. The average length of employment was 12 years.

### Instruments and measures

Musculoskeletal pain was measured by questions from the Subjective Health Complaints questionnaire (SHC) (Eriksen et al., 1999) asking about the severity of musculoskeletal pain in a 4-point rating scale; ‘none’ (0), ‘some’ (1) ‘much’ (2), and ‘severe’ (3). Musculoskeletal pain from the upper body was measured by four items including shoulders, arms, neck, upper back (*α* = 0.72), while musculoskeletal pain from the lower back was measured by one item.

We measured physical activity with the item: “for how many hours, an ordinary week, do you perform hard physical activity (sweating/out of breath)?”. The item was selected as it is included in several large-scale health studies and has been shown to be a valid and reliable measure of self-reported physical activity (Kurtze et al., 2007).

The general stress at work was measured with a validated single-item measure (Elo et al., 2003; Houdmont et al., 2019) with wording as follows: “Stress means a situation in which a person feels tense, restless, nervous or anxious or is unable to sleep at night because his/her mind is troubled all the time. Do you feel this kind of stress these days?”. Response categories were ranging from ‘Not at all’ (1) to ‘Very much’ (5).

Organisational work support was measured with five items from the general Nordic Questionnaire for Psychological and Social Factors at Work (QPS Nordic) (Dallner et al., 2000). Three items focused on support from leader: ‘If needed, can you get support and help with your work from your immediate superior?’, ‘If needed, is your immediate superior willing to listen to your work-related problems?’, and: ‘Are your work achievements appreciated by your immediate superior?’. Two items addressed colleague support: ‘If needed, can you get support and help with your work from your colleagues?’ and ‘If needed, are your co-workers willing to listen to your work-related problems?’ Response categories ranged from ‘Very seldom’ (1) to ‘Very often or always’ (5). Cronbach’s alpha was α = 0.89.

### Data analysis

We used IBM Statistical Package for the Social Sciences (IBM SPSS) version 28.0 for the analysis. In adittion, G*power 3 (Faul et al., 2007) was used to estimate the statistical power of the study. Pearson’s correlation coefficient (*.r*) was used to explore the associations between variables. Multiple hierarchical, regression analysis was used to investigate whether organisational work support predicted lower and/or upper musculoskeletal pain controlling for general stress level.

A power analysis indicated that the sample size was marginally sufficient. To ensure a power > .80, the predictors in the regression models were kept to a minimum. According to Cohen (1992), the values of .*r* equal respectively 0.10, 0.30, and 0.50 represent small, medium, and large effect sizes.

## Ethical approval

This study was approved by the Regional Committee for Medical and Health Research Ethics (REK, 2019/7168).

## Results

A majority of the participants (71%, *n* = 62) reported that they were exercising more than 2 hours a week. With a mean score of over 4 hrs (4.3, SD 2,73) of exercise weekly, this sample report to be more physically active than other comparable populations (Fanavoll et al., 2016; Jonsdottir et al., 2010; Kurtze et al., 2003; Solbraa et al., 2015).

In this sample (*N* = 77), 23 (29.9%), reported much or severe musculoskeletal pain (>2) from the neck, 16 (20.8%) reported much or severe lower back pain, 15 (19.5%) reported upper back pain, and 13 (16.9%) reported pain from shoulders. Only two reported much or severe musculoskeletal pain from arms.

Thirty-two percent (*n* = 25) of the respondents reported “no stress”, 54% (*n* = 42) reported a little or some stress, while 10 participants (13%) reported much or very much stress. Stress was significantly positively correlated with upper musculoskeletal pain (0.30) and negatively correlated with support (−0.34, *p* < .01). Stress was not associated with lower back musculoskeletal pain. Comparing this sample to a large (*n* = 1133) national cross-sectional study of employees in the Norwegian Police Service (Langvik et al., 2021), showed a significantly higher level of stress in this group *t* (76) = 2.12, *p* = .02, *d* = 1.0 representing a large effect-size (Cohen, 1992).

We hypothesised that musculoskeletal pain was negatively associated with physical activity (H1). Contrary, physical activity had no significant correlation with either perceived stress, support, or musculoskeletal back pain, and was therefore excluded from further analysis. Table 1 presents the descriptive for the variables included in the study and the associations between them.

**Table 1.** Descriptives and associations between variables (.*r*).

*N* = 77	M (SD)	1	2	3	4	5	6
1. Lower back pain	1.77 (0.84)						
2. Upper back, shoulder, and arm pain	1.63 (0.55)	0.28*					
3. Physical activity	4.31 (2.74)	−0.09	−0.06				
4. Work support	4.03 (0.98)	−0.24*	−0.28*	0.07			
5. Stress	2.18 (1.08)	0.08	0.31**	0.09	−0.34**		
6. Age (median)	“30–39”	0.03	0.22	−0.07	−0.21*	−0.01	
7. Gender (female)	49.4%	0.13	0.21	0.06	−0.01	0.16	0.06

*p < .05; **p < .01.

We hypothesised that musculoskeletal pain was negatively associated with support (H2) and positively associated with stress (H3). Tables 2 and 3 show the results from the multiple regression analysis predicting musculoskeletal pain in the upper back (Table 2) and in the lower back (Table 3) respectively. In both analyses, we included organisational work support as a predictor in model 1, controlling for age and gender, while adding stress in model 2.

**Table 2.** Predictors of upper musculoskeletal back pain.

	Model 1		Model 2	
	*β*	SE	*β*	SE
Work support	−0.24*	0.16	−0.16	0.16
Gender	0.20	0.27	0.16	0.27
Age	0.17	0.24	0.19	0.23
Stress			0.23*	0.23
	*R*^2^(*adj*) = 15 (*12*)		*R*^2^(*adj*) = 20 (*15*)	

*p < .050; **p < .01.

**Table 3.** Predictors of lower back musculoskeletal pain.

	Model 1		Model 2	
	*β*	SE	*β*	SE
Work support	−0.26*	0.06	−0.27*	0.07
Gender	0.14	0.11	0.14	0.11
Age	−0.09	0.10	−0.09	0.10
Stress			−0.04	0.09
	R^2^(*adj*) = 8(*5*)		*R*^2^(*adj*) = 9(*3*)	

*p < .05.

Table 2, shows that 15% of the variance in upper musculoskeletal back pain was accounted for by organisational work support, age, and gender F(3,73) = 4.30, *p* = .008 in model 1. Organisational work support (*β* = −0.24, *t* = −2.02, *p* = .03) was a significant predictor of upper muscular pain while the remaining predictors were not. When adding ‘stress’ in Model 2, the total variance explained increased significantly (*F* Change = 4.10, *p* .047) to 20%, and stress was the only significant predictor of upper musculoskeletal pain (*t* = 2.02, *p* = .047) in model 2 (*F*(4,72) = 4.38, *p* =.003).

Table 3, shows that 8% of the variance in lower back musculoskeletal pain was accounted for by the organisational work support, age, and gender in Model 1 (F(3,73) = 2.21, *p = .09*). Organisational work support was a significant predictor of lower back musculoskeletal pain, (*t* = −2.26, *p* = .027). Adding ‘stress’ to the predictors in Model 2 did not increase the variance explained, and stress was not a significant predictor of lower back pain in Model 2. Organisational work support was a significant predictor of lower back pain when controlling for age, gender, and stress. However, the model in total was not significant (*F* (4,72) = 1.66, *p = .17*).

## Discussion

Drawing upon the JD-R model on how the interplay between job demands and job resources are associated with job stress, health, and well-being, this study explored the associations between musculoskeletal pain and physical activity (H1), organisational work support (H2), and prevalence of musculoskeletal pain controlled for stress (H3) among police investigative interviewers working in special victim units. While former studies mainly have focused on how work demands associated with “traditional” forms of police work are related to ill-health (e.g., burnout) (Bakker and Demerouti, 2017; Wolter et al., 2019), this study explored musculoskeletal pain among police investigative interviewers, another and equally important police work task. The health and well-being of this police workgroup of highly skilled employees in the police are central, as demanding psychosocial working conditions and lack of buffering factors may increase sickness absence, which poses a threat to the legal protection of involved third parties, in regard to quality and quantity (e.g., high turnover).

Similar to other studies in the police (e.g., Colgan et al., 2019), and worldwide (Hartvigsen et al., 2018), our sample reported relatively high levels of musculoskeletal pain. The police officers in our study scored higher on perceived stress compared to other police employees (Langvik et al., 2021). However, only 13% reported much or very much stress, which is lower compared to other occupations (Skogstad, 2001). In the regression analyses, musculoskeletal back pain was negatively associated with organisational work support. For upper back pain, support was not a significant predictor when controlled for stress. Contrary to our hypothesis, based on the documented health benefits (Bull et al., 2020), and the knowledge of the curative effect of physical activity in the occurrence of musculoskeletal pain (see e.g., Van Hecke et al., 2013), we found no associations between physical activity and neither musculoskeletal pain nor levels of stress among police investigative interviewers. The high level of regularly physical activity of over 4 hours weekly in this sample delimits generalisation to a more general population. However, other studies on stress and physical activity in police offices have observed no associations between physical activity and stress (Ramey et al., 2014), supporting our finding.

Studies on police stress indicate that organisational job demands take a greater toll on police officers’ health than operational job demands (Rabbing et al., 2022; Shane, 2020; Wolter et al., 2019). Employees’ alignment with their organisation’s strategic objectives is highly connected to wellbeing and engagement (Biggs et al., 2014). Within the JD-R framework, job demands such as emotional efforts and general high workload may be alleviated by both organisational job resources such as autonomy, social support, quality of relationship with supervisor, performance feedback, and by individual resources such as a health-promoting lifestyle (Bakker and Demerouti, 2017). Systematic moderate-intensity physical activity has been found to prevent negative emotions (Long et al., 2021). On the other hand, among chronically stressed populations, such as law enforcement officers, stress and physical activity may have an inverse relationship where perceived stress hinders physical activity (Stults-Kolehmainen and Sinha, 2014).

Perceived stress is a strong predictor of upper musculoskeletal pain in our sample. Interestingly, stress was not a significant predictor of lower back pain, only of upper musculoskeletal pain. This suggests that the relationship between pain and stress is not general, and that specific bodily areas, like the neck and shoulders, are more vulnerable to stress. The recent study by Zare and colleagues (2021), found that job stress, time pressure, and insufficient work support significantly related to the onset of upper musculoskeletal back pain among nurses, hence supporting our finding.

Perceived organisational work support from leaders and colleagues, was a significant predictor of musculoskeletal pain in our study. The work conducted by police investigative interviewers often involves interviews with traumatized persons and/or persons exposed to trauma (Jakobsen et al., 2017; Risan et al., 2020). These work tasks require skills in gathering sufficient information and detailed accounts from an interviewee while establishing and maintaining rapport (Jakobsen et al., 2017; Risan, 2017; Risan et al., 2016a, 2016b). However, the performance by of the investigative interviewers do not solely depend on their individual skills, but also on support from supervisors. An individual component of leadership is determined by the way leaders act towards their employees (Engel et al., 2018), and supportive leadership is central for the police employees health and well being (Sørengaard and Langvik, 2022). Investigative interviewers report supervisors undermining the opportunities of developing and maintaining good interview techniques, contributing to high working demands, such as high case- and workload, as well as to expectations of invulnerability (Iversen, 2021; Risan et al., 2016a), the latter explained by police culture values such as toughness, self-reliance, and suppressing weaknesses (Soomro and Yanos, 2019). Obstructions in developing employees’ interviewing skills due to caseload and generally high workload counteract organizational goals and weaken the professional alignment which is one of the main reasons for strain in the JD-R model. On the other hand, social interaction, and organisational work support from colleagues and leaders mitigates demands at work, decreases health complaints (Johnsen et al., 2018; McCanlies et al., 2018) and promotes a positive working climate (Wolter et al., 2019). The organisational resources should encourage sufficient supervision and colleague support. Organisational work support from supervisors in the form of appreciation for employees' special skills and competence, recognising the emotional efforts during interviews, and facilitating the opportunity for social interactions at work, may function as a buffer against adverse health (Wolter et al., 2019). Mandatory education and professional group training, both for supervisors and employees, could be an avenue for improving supervisory skills, working climate, and employees’ resilience to emotional demands at work.

## Strength and limitations

The small sample and the cross-sectional design are limitations in this study. A larger sample would also allow for including more predictors. However, estimation of statistical power suggests that the power was sufficient for the exact analysis performed. A strength of this study is the use of paper-and-pen surveys, assuring the respondents’ anonymity, and the use of validated instruments. Self-reported information on physical activity and sedentary behaviour should be used with caution among groups reporting back pain (Gupta et al., 2018). Even though there is no gold standard for measuring physical activity (Nigg et al., 2020), the measurement used may have shortcomings.

## Implications and suggestions for further research

In this study, we explored the prevalence of musculoskeletal pain and stress among investigative interviewers. The results suggest that organisational work support from colleagues and leaders should be given more attention in this workgroup. Our results also provide interesting insight into the prevalence of musculoskeletal back pain among police investigative interviewers and possible preventive associations of importance.

Our results illustrate that the association between stress and pain not necessarily is alleviated by physical activity when the latter is high. This supports previous studies finding conflicting results on the contribution of psychosocial factors to the onset and persistence of chronic pain (Abdallah and Geha, 2017; Bonzini et al., 2015), and should be investigated further.

## Conclusion

Organisational work support from leaders and colleagues and stress was significant correlated to musculoskeletal back pain, whereas physical activity was not. Perceived stress was a significant predictor of upper, but not lower musculoskeletal back pain. Perceived organisational work support from leaders and colleagues, was a significant predictor of lower, but not upper body musculoskeletal pain. The police investigative interviewers in this study reported higher levels of stress than other police employees. A more nuanced understanding of the interplay between demands and resources for this unique and highly specialised workgroup warrants more research.
